# Solar-powered bioelectrochemical system for efficient cadmium remediation and recovery of reusable solids

**DOI:** 10.1039/d5ra07856c

**Published:** 2026-01-14

**Authors:** Yeonu Im, Minsoo Kim, Yuri Kim, Soo Youn Lee, Minkyoung Kim, Tae-Hoon Kim, Jinhee Heo, Changman Kim

**Affiliations:** a Department of Biotechnology and Bioengineering, Chonnam National University Gwangju 61188 Republic of Korea cmkim@jnu.ac.kr +82 62 530 1949 +82-10-4566-2661; b School of Chemical Engineering, Pusan National University Busan 46241 Republic of Korea; c Gwangju Bioenergy R&D Center, Korea Institute of Energy Research (KIER) Gwangju 34129 Republic of Korea; d Department of Oceanography/KNU G-LAMP Project Group, Kyungpook National University Daegu 41566 Republic of Korea; e Department of Earth Systems and Environmental Sciences, Chonnam National University Gwangju 61188 Republic of Korea; f Advanced Characterization & Analysis Research Group, Korea Institute of Materials Science (KIMS) Changwon 51508 Republic of Korea; g Institute of Synthetic Biology for Carbon Neutralization, Chonnam National University Gwangju 61188 Republic of Korea

## Abstract

Cadmium (Cd) is a persistent industrial pollutant that poses severe environmental risks, necessitating remediation technologies that balance efficiency with energy sustainability. This study investigates a solar-powered bioelectrochemical system (PV-BES) as a low-energy strategy for Cd recovery, addressing limitations associated with conventional energy-intensive processes. In batch experiments, the PV-BES achieved 62.3% removal of dissolved Cd^2+^, outperforming the 53.8% efficiency of a self-biased microbial fuel cell. Electrochemical measurements combined with solid-phase characterization indicate that the solar-driven bias is consistent with localized interfacial alkalization, which favors the formation of crystalline Cd–O solids rather than continuous metallic Cd plating under near-neutral bulk pH conditions. These results suggest that coupling photovoltaics with bioanodes provides a synergistic, reagent-free pathway for cadmium immobilization and recovery. Overall, this work supports the potential of PV-assisted BES as a sustainable, low-energy approach for heavy metal remediation under mild aqueous conditions.

## Abstract 2

A solar-powered bioelectrochemical system integrates a photovoltaic bias with a bioanode to enable cadmium remediation and recovery under mild aqueous conditions, favoring precipitation of Cd–O solids over metallic deposition.

## Graphical abstract

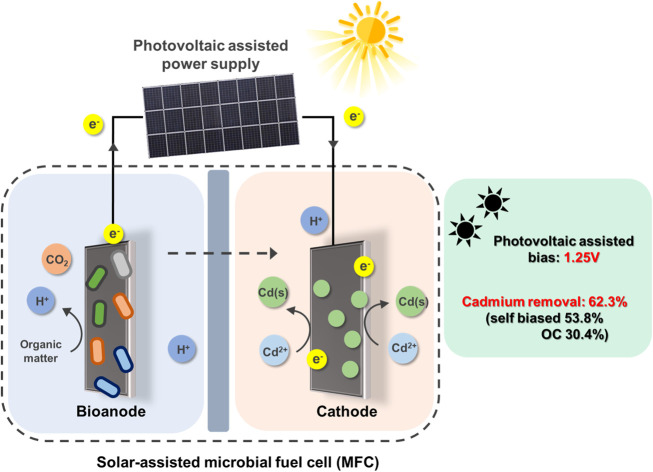

## 1 Introduction

Cadmium (Cd) is a widely used but highly hazardous metal.^1,2^ Because of its persistence and carcinogenicity, Cd is regulated as a priority pollutant.^3–5^ Industrial releases and legacy contamination continue to cause serious ecological and human health risks, including kidney dysfunction, skeletal damage, and cancer.^6,7^ Anthropogenic activities can elevate Cd concentrations far beyond background levels.^8,9^ This makes effective removal and recovery from Cd-bearing effluents an environmental and resource priority.^10^

In water, Cd occurs predominantly as mobile Cd^2+^, and its fate is governed mainly by pH and redox conditions. The World Health Organization (WHO) guideline value for Cd in drinking water is 0.003 mg L^−1^.^11,12^ Conventional treatments (*e.g.*, precipitation, adsorption, membrane separations, and electrowinning) can remove Cd,^13^ but they often require strict pH control and intensive chemical/energy inputs and generate secondary wastes.^14^ Electrowinning typically requires acidic electrolytes and high applied voltages;^15^ membranes are costly and energy-intensive;^16^ and precipitation produces large sludge volumes and performs poorly at low concentrations.^17,18^ To mitigate these limitations, recent studies have explored diverse biotechnological strategies, including nanobioremediation,^19^ endophytic bacteria,^20^ extremophilic algae,^21^ and advanced nanocomposites.^22^

Among emerging alternatives, bioelectrochemical systems (BES) couple microbial oxidation with cathodic electrochemical reactions and can reduce external chemical demand for metal removal and recovery.^23–26^ Despite these advantages, cadmium recovery presents distinct challenges. The standard reduction potential of the Cd^2+^/Cd^0^ couple (−0.40 V *vs.* SHE) is relatively negative, making direct Cd plating difficult at the modest voltages typically produced in self-biased MFCs.^27^ Due to these constraints, Cd in BES is often immobilized as precipitated solids rather than recovered as metallic deposits, while competing cathodic reactions (*e.g.*, hydrogen evolution) further limit recovery efficiency.^4^ A critical knowledge gap remains as to whether a modest, renewable electrical bias can systematically shift cadmium recovery pathways in bioelectrochemical systems under mild aqueous conditions. We hypothesized that photovoltaic (PV) assistance would provide additional cathodic driving force, enhance proton-consuming reactions, and induce localized alkalization, thereby promoting precipitation-dominated recovery of oxygen-containing Cd solids under mild bulk pH conditions. To test this hypothesis, we developed a PV-assisted BES integrating a bioanode, a membrane separator, and a planar indium tin oxide (ITO) cathode, and compared open-circuit, self-biased, and PV-assisted modes. Dissolved Cd^2+^ was quantified by ICP-OES, recovered phases were characterized by SEM/EDS/XRD, and electrochemical measurements with thermodynamic interpretation (Pourbaix) were used to elucidate bias-dependent recovery pathways.

## 2 Experimental

This section outlines the materials, reactor configuration, and operating conditions used to evaluate the effect of photovoltaic-assisted bias on cadmium recovery pathways in a dual-chamber bioelectrochemical system.

### 2.1. Chemicals and synthetic solutions

All reagents were of analytical grade and used without further purification. Deionized (DI) water (18.2 MΩ cm) was used for all solution preparations. The cadmium stock solution was prepared by dissolving CdCl_2_·2.5H_2_O (98.0%, Kanto Chemical Co, Japan) in DI water and diluting to approximately 1000 mg per L Cd^2+^ to serve as the initial working catholyte.

The anolyte composition (per liter of DI water) was as follows: glucose (5.0 g, 99.5%, Sigma-Aldrich, USA), K_2_HPO_4_ (5.36 g, 98.0%, Duksan Chemicals, Korea), KH_2_PO_4_ (2.62 g, 99.0%, Sigma-Aldrich, USA), NH_4_Cl (0.23 g, 99.5%, Daejung Chemicals, Korea), NaCl (0.80 g, 99.5%, Daejung Chemicals, Korea), MgSO_4_·7H_2_O (0.20 g, 98.0%, Sigma-Aldrich, USA), and yeast extract (0.50 g, Daejung Chemicals, Korea). Anaerobic activated sludge obtained from the Korea Environmental Corporation (Republic of Korea) was used to inoculate the anode. The inoculum was pre-enriched through successive 7-day transfers over two months to establish a reproducible electroactive microbial consortium. Enrichment was performed in the same medium, with nitrogen sparging for 10 min prior to sealing to maintain anaerobic conditions.

### 2.2. Reactor configuration

**Fig. 1:**
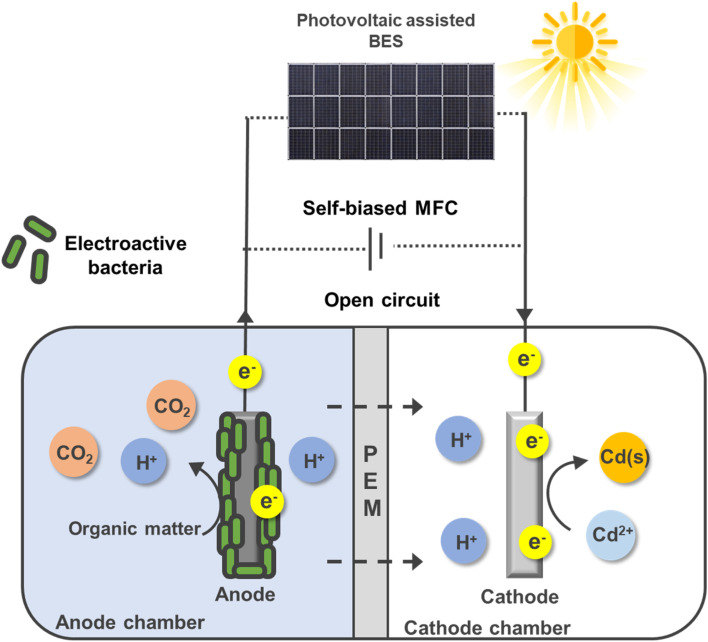
Dual-chamber solar-assisted BES showing the bioanode, proton-exchange membrane, and ITO cathode.

Experiments were conducted in dual-chamber glass bioelectrochemical cells, each with a working volume of 250 mL per chamber. The chambers were separated by a proton-exchange membrane (Nafion 212, Chemours, USA). The anode consisted of commercial carbon felt (4 × 5 cm, 0.6 mm thick; Fuel Cell Store, USA) fully submerged in the anolyte. The cathode was indium tin oxide (ITO)-coated glass (1500 Å ITO on 0.5 mm soda–lime glass; IMAT, Republic of Korea), cut into 2 × 4 cm and 2 × 6 cm sections, with an effective immersed surface area of 2 × 3 cm in all experiments. Electrical connections were established using silver paste, and exposed areas were sealed with epoxy to prevent electrolyte leakage and corrosion of leads. The side arms were sealed with butyl rubber stoppers and aluminum crimps (three ports for the anode, two for the cathode). The anode chamber was inoculated with anaerobic activated sludge and operated as a mixed microbial consortium. Unless otherwise stated, all tests were conducted at room temperature (25 ± 2 °C). A schematic illustration of the dual-chamber photovoltaic-assisted bioelectrochemical reactor, including the anode, cathode, proton-exchange membrane, and external PV connection, is shown in Fig. 1.

### 2.3. Operating modes

Three operating modes were evaluated using identical dual-chamber reactors. In the open-circuit (OC) mode, electrodes were immersed without electrical connection. In the self-biased microbial fuel cell (MFC) mode, the anode and cathode were connected through a 1 kΩ external resistor, allowing current generation by microbial metabolism. In the solar-assisted BES mode, a 60 × 80 mm photovoltaic panel was connected in series with the cell and operated under a controlled laboratory spotlight, providing a stable external bias of approximately 1.25 V selected to enhance the cathodic driving force for cadmium conversion while avoiding excessive parasitic reactions.

In all configurations, the bioanode functioned as the positive electrode (electron source) and the ITO cathode as the negative electrode (electron sink). Each batch cycle lasted seven days, after which both the anolyte and the Cd-bearing catholyte were replaced with fresh media for subsequent cycles. A planar ITO cathode was intentionally selected for its chemical inertness, sufficient electrical conductivity, and optical transparency, which enable direct surface and phase characterization of recovered cadmium species without interference from porous substrates. The photovoltaic (PV) panel was operated under controlled indoor laboratory conditions rather than outdoor solar irradiation. A laboratory spotlight was used to continuously illuminate the PV panel for 24 h per day, providing a stable voltage bias throughout the experimental period. All experiments were conducted in a temperature-controlled environment maintained at 25 ± 3 °C. Because the reactor system was closed and the PV was operated indoors under constant illumination, external weather conditions (*e.g.*, humidity, rainfall, or seasonal variation) did not influence the electrical output during the 7-day batch operation. In this study, the PV module was used as a voltage source to supply a controlled bias rather than being operated at its maximum power output.

### 2.4. Sampling and analysis

Catholyte samples (∼5 mL) were collected at day 0 and 7 from quiescent reactors without stirring. Supernatants were decanted carefully and filtered through 0.2 µm syringe filters. The filtrates were stored at 4 °C until analysis. Dissolved Cd^2+^ concentrations were quantified using inductively coupled plasma optical emission spectroscopy (ICP-OES; iCAP 7400 Duo MFC, Thermo Fisher Scientific, USA) with matrix-matched standards.

Cadmium removal efficiency (%) was calculated according to eqn (1):1
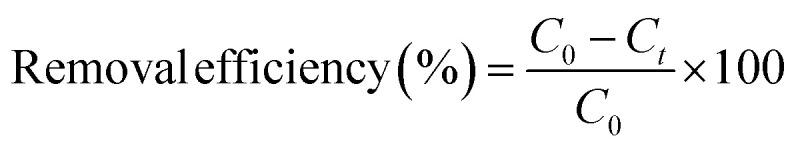
where *C*_0_ and *C*_*t*_ represent the initial and day-7 Cd^2+^ concentrations in the filtered supernatant, respectively. Unless otherwise noted, all data represent the mean ± standard deviation (SD) of three independent batch runs (*n* = 3), with replicate numbers indicated in the figure captions. Cyclic voltammetry (CV) measurements were conducted using the ITO cathode as the working electrode, an Ag/AgCl reference electrode, and a platinum wire counter electrode at room temperature.

### 2.5. Cathode characterization

Following each experimental run, ITO cathodes were gently rinsed with DI water, air-dried, and analyzed by scanning electron microscopy (SEM; JEOL JSM-IT300) equipped with energy-dispersive X-ray spectroscopy (EDS; Oxford Aztec Energy X-Max, 50 mm^2^). Pristine ITO was used as control. Crystalline phases were identified by X-ray diffraction (XRD; Rigaku SmartLab 9 kW, Cu Kα source). Air-dried precipitates collected from the catholyte were also subjected to XRD analysis.

### 2.6. Thermodynamic and kinetic analyses

A simplified Pourbaix diagram for the Cd–H_2_O system (25 °C) was constructed to qualitatively interpret potential-dependent transformations, including Cd^2+^/Cd^0^ and Cd(OH)_2_/CdO equilibria, in relation to water stability boundaries (H^+^/H_2_ and O_2_/H_2_O). Activities were estimated from initial solution compositions, and complexation beyond hydroxide and carbonate species was not explicitly modeled. To further examine reaction competition, Butler–Volmer (BV) analyses were conducted to compare Cd^2+^ reduction kinetics with the hydrogen evolution reaction (HER) on ITO surfaces. Representative kinetic parameters and diffusion-limited currents were used to generate interpretive BV plots, which are provided in the Supporting Information. These plots were intended for qualitative interpretation rather than quantitative fitting.

## 3 Results and discussion

### 3.1. System validation and operating modes

A dual-chamber, bioanode-driven cell was constructed to maintain a stable electron supply while preventing oxygen evolution at the anode during polarized operation (Fig. 1). The enriched bioanode exhibited consistent performance across replicate batches, confirming reliable biological activity. The three operating modes performed as designed: the open-circuit (OC) configuration provided no external current; the self-biased MFC generated a moderate potential across a 1 kΩ external resistor; and the solar-assisted mode applied an additional bias of approximately 1.25 V using a compact PV panel. Polarity was fixed in all cases, with the bioanode serving as the positive electrode and the ITO cathode as the negative electrode. All tests were conducted under quiescent conditions in 7-day batch cycles, as detailed in Section 2. This configuration enabled direct comparison of cadmium transformation pathways under identical physicochemical conditions but with distinct electrochemical driving forces.

### 3.2. Reduction-driven Cd removal from the supernatant

**Fig. 2:**
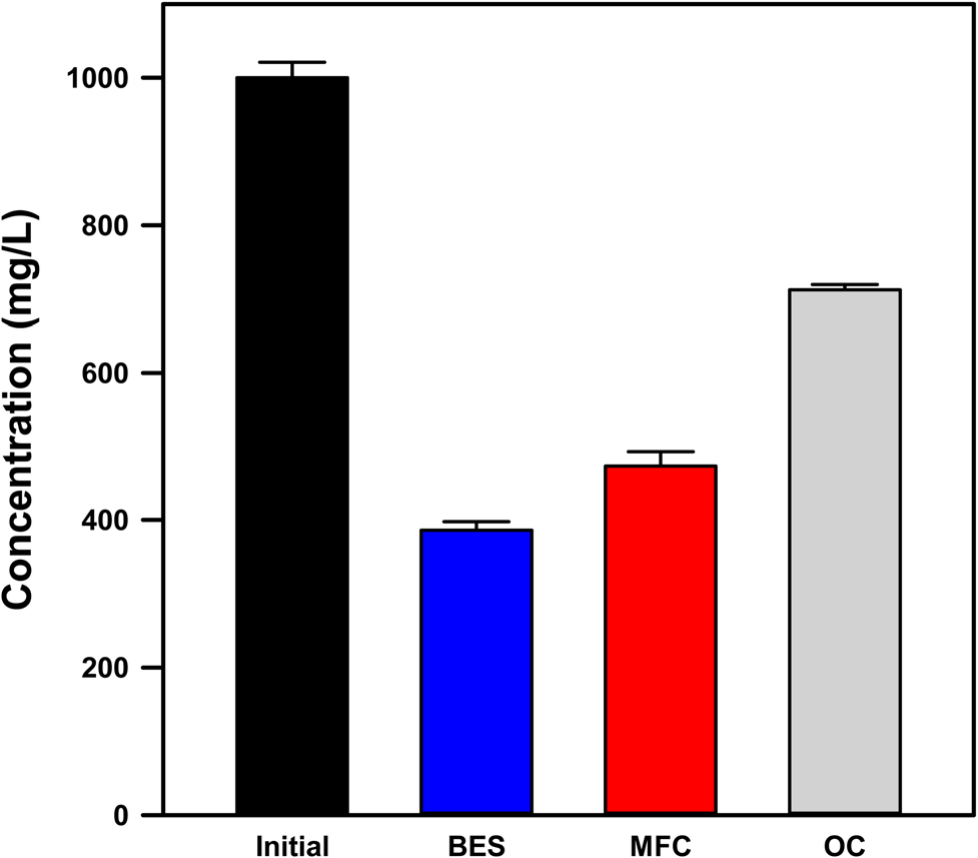
Dissolved Cd concentrations after seven days under OC, MFC, and solar-assisted BES conditions. Data represent mean ± SD (*n* = 3).

Cadmium removal was quantified using filtered (0.2 µm) supernatant samples from quiescent reactors to exclude electrode-associated and settled particulates. Therefore, “removal” here refers specifically to the conversion of Cd from the dissolved (<0.2 µm) fraction rather than total mass removal. The initial Cd^2+^ concentration was 1023.0 ± 11.1 mg L^−1^. After 7 days (Fig. 2), the solar-assisted BES achieved a final dissolved Cd concentration of 385.99 ± 1.15 mg L^−1^ (62.3% removal), compared with 473.07 ± 1.97 mg L^−1^ (53.8% removal) in the self-biased MFC and 712.32 ± 7.32 mg L^−1^ (30.4% removal) in the OC control.

The removal trend (BES > MFC > OC) clearly demonstrates a dependence on the applied bias. The photovoltaic-assisted bias enhanced the cathodic overpotential and proton consumption at the ITO surface, which facilitated Cd reduction and immobilization relative to the self-biased mode. Over the 7-day period, the solar-assisted BES removed approximately 87 mg L^−1^ more Cd than the MFC, corresponding to a 15.8% higher removal efficiency, and achieved roughly double the removal observed in the OC control (62.3% *vs.* 30.4%). These results represent removal from the dissolved fraction only (<0.2 µm) and thus capture electrochemically driven immobilization processes rather than total Cd mass balance. This study focuses on changes in the dissolved cadmium fraction to elucidate bias-dependent recovery pathways, while a full phase-resolved cadmium mass balance (dissolved, settled, and electrode-bound fractions) is identified as an important direction for future work.

### 3.3. Surface characterization and phase evolution

**Fig. 3:**
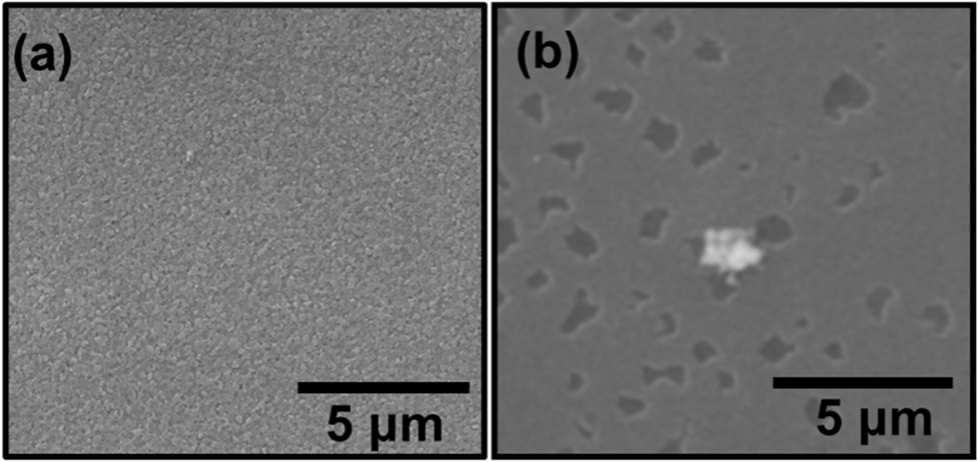
SEM micrographs of ITO: (a) pristine and (b) after solar-assisted BES operation.

Microscopic and compositional analyses provided insights into the fate of cadmium removed from solution. The pristine ITO surface appeared smooth and featureless at the micrometer scale (Fig. 3a). After operation in the solar-assisted BES, the cathode surface exhibited sparse, island-like particulates distributed over an otherwise clean background (Fig. 3b). The absence of a continuous film suggests that most Cd was immobilized near the cathode interface and subsequently detached as suspended or settled precipitates rather than forming a coherent electrode-bound layer.

**Fig. 4:**
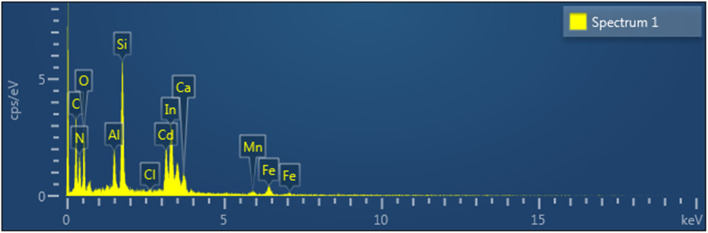
EDS mapping and spectrum of a representative surface particle on the ITO cathode. Quantitative composition is summarized in Table 1.

EDS was conducted on representative surface particulates to determine their elemental composition (Fig. 4 and Table 1). The spectrum displayed distinct Cd peaks accompanied by oxygen and minor chloride signals, along with In and Si originating from the ITO-glass substrate. Trace elements such as C, Al, Ca, Mn, and Fe were also detected, likely due to sample mounting media or residual constituents from the electrolyte. Quantitatively, the analyzed area contained 10.94 wt% Cd, 24.08 wt% O, 14.62 wt% In, and 8.80 wt% Si (Table 1). Because the electron interaction volume included both the surface deposit and underlying substrate, the reported Cd fraction represents a conservative estimate of the deposit composition. The strong oxygen signal, together with minor chloride detection, indicates that the adherent features correspond to oxygen-containing Cd deposits incorporating traces of electrolyte-derived species. The relatively high C and N signals detected by EDS are attributed to sample mounting materials and residual electrolyte components, as well as contributions from the underlying substrate within the electron interaction volume, and are therefore not interpreted as intrinsic constituents of the cadmium deposits.

**Table 1:** Quantitative EDS composition of the post-run ITO cathode following Cd recovery in the solar-assisted BES experiment. C and N signals are considered artefactual and are not included in the interpretation of cadmium deposit composition. The absolute Cd wt% values should be interpreted qualitatively, as the electron interaction volume includes contributions from both the surface deposit and the underlying ITO-glass substrate

Element	wt%	wt% Sigma	Atomic %
C	20.44	1.44	34.82
N	13.08	2	19.1
O	24.08	1.18	30.8
Al	2.71	0.19	2.06
Si	8.8	0.36	6.41
Cl	0.27	0.09	0.16
Ca	1.35	0.19	0.69
Mn	0.79	0.2	0.29
Fe	2.92	0.29	1.07
Cd	10.94	0.6	1.99
In	14.62	0.69	2.61
Total	100		100

**Fig. 5:**
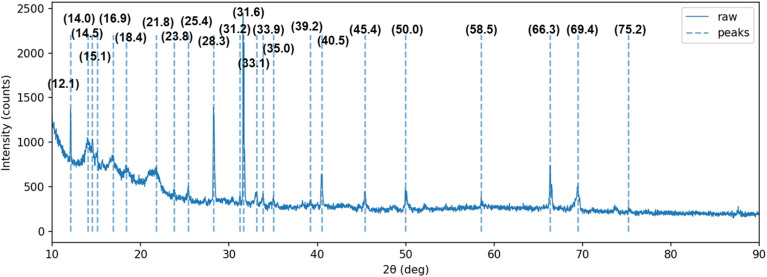
Powder XRD pattern of Cd-bearing precipitates collected from the PV-assisted BES catholyte.

Complementary XRD analysis was performed on visible particles collected from the catholyte after drying (Fig. 5). The diffractogram exhibited sharp reflections at 2*θ* ≈ 31.6°, 39.2°, 45.4°, 50.0°, 66.3°, 69.4°, and 75.2°, characteristic of Cd–O solids such as CdO and/or dehydrated derivatives of (Cd(OH)_2_). Additional low-angle features likely originated from co-precipitated salts. Taken together, these results—sparse adhesion on ITO, Cd/O-rich composition on the cathode surface, and crystalline Cd–O phases in the collected precipitates—indicate a precipitation-dominated immobilization pathway. In this process, Cd nucleation occurs at the cathode interface, followed by particle detachment and settling in the bulk catholyte. This product distribution explains the substantial decrease in dissolved Cd concentration observed in Section 3.2 without the formation of a thick adherent film. The findings further support the bias-dependent mechanism discussed in Section 3.4.

### 3.4. Mechanistic interpretation and bias-dependent pathways

**Fig. 6:**
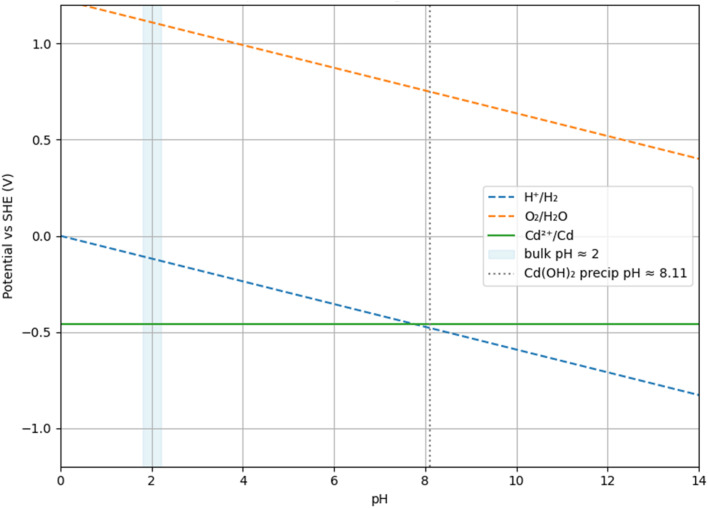
Simplified Pourbaix diagram for Cd–H_2_O at 25 °C, illustrating bias- and pH-dependent domains for Cd^0^ and Cd-oxy(hydroxide) solids.

The observed removal hierarchy (BES > MFC > OC) reflects the influence of cathodic potential and the near-electrode microenvironment on Cd immobilization. Under PV assistance, the applied bias enhances electron transfer to Cd^2+^ and drives proton-consuming reactions (*e.g.*, hydrogen evolution and, where oxygen is available, oxygen reduction), which is consistent with the development of a locally alkaline microenvironment at the cathode interface, relative to the near-neutral bulk solution (Fig. 6). The initial bulk pH of the catholyte was 5.65, and after 7 days of operation the bulk pH was 5.27 ± 0.15 in the PV-assisted BES and 5.68 ± 0.05 in the self-biased MFC (mean ± SD, *n* = 3; Fig. S1). In this regime, two cadmium immobilization pathways are thermodynamically plausible under these conditions: (i) direct electrodeposition of Cd^0^ when the local potential exceeds the Cd^2+^/Cd^0^ threshold (≈−0.40 V *vs.* SHE), and (ii) interfacial precipitation of oxygen-containing Cd solids (*e.g.*, Cd(OH)_2_), which can dehydrate to CdO as the local pH increases.

**Fig. 7:**
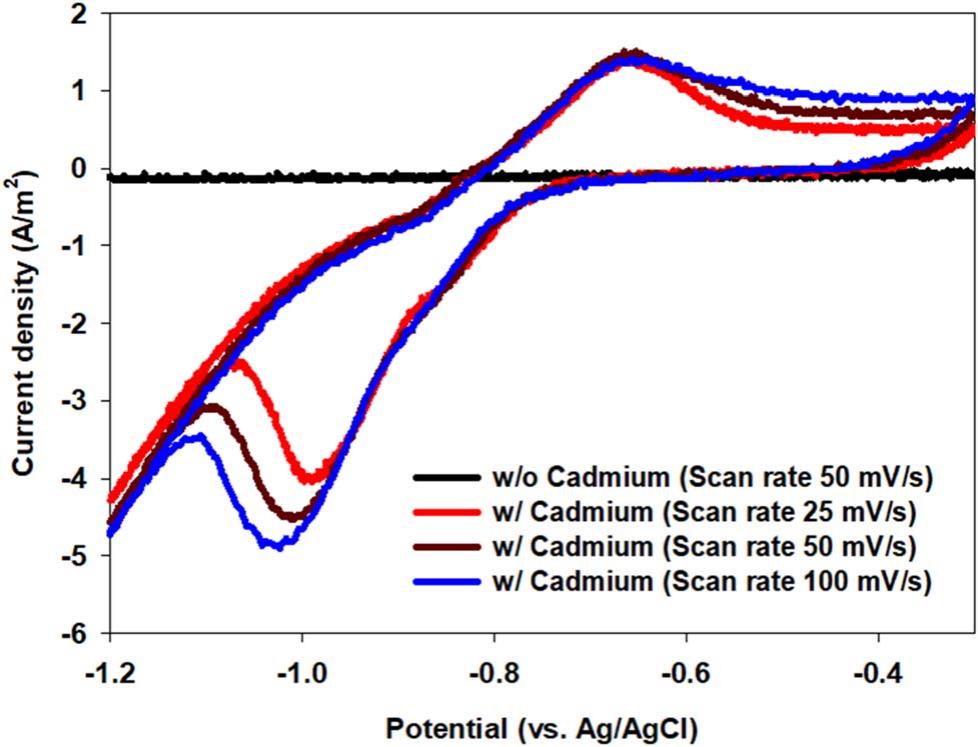
Cyclic voltammograms of the ITO cathode recorded in Cd^2+^-containing electrolyte using an Ag/AgCl reference electrode under controlled laboratory conditions. Enhanced cathodic activity under PV-assisted bias and the absence of a pronounced anodic stripping peak indicate precipitation-dominated cadmium immobilization rather than continuous metallic Cd deposition.

The sparse islands observed on the ITO surface (Fig. 3b), the Cd and O signals in EDS (Fig. 4 and Table 1), and the crystalline Cd–O reflections in XRD (Fig. 5) together indicate that precipitation-dominated immobilization is the prevailing pathway under the tested conditions, consistent with the near-neutral bulk pH and the proposed localized interfacial alkalization mechanism (see also Fig. 7).

To provide experimental electrochemical evidence supporting the proposed recovery pathways, cyclic voltammetry (CV) was performed using the ITO cathode in Cd^2+^-containing electrolyte with an Ag/AgCl reference electrode (Fig. 7). In the absence of an applied bias, the CV response exhibited limited cathodic activity. Under PV-assisted conditions, enhanced cathodic currents were observed in the potential region relevant to Cd^2+^ reduction and proton-consuming reactions, consistent with increased electrochemical driving force at the cathode interface. Notably, no pronounced anodic stripping peak characteristic of continuous bulk metallic Cd deposition was observed, supporting a precipitation-dominated cadmium immobilization pathway.

In the self-biased MFC, the smaller overpotential limits nucleation rates and interfacial alkalization. Cadmium conversion still occurs (Fig. 2) but to a lesser extent over seven days. In the OC control, only slow, non-faradaic processes contribute, resulting in minimal removal. The simplified Pourbaix diagram (Fig. 6) qualitatively captures these trends: at near-neutral bulk pH, deeper cathodic polarization shifts the interface from the dissolved Cd^2+^ domain into regions where Cd^0^ and Cd-oxy(hydroxide) solids are thermodynamically favored. Consistent with this interpretation, Butler–Volmer competition analysis (Fig. S2) predicts that PV-assisted conditions favor Cd reduction over the hydrogen evolution reaction by roughly an order of magnitude, explaining the enhanced removal of dissolved Cd without requiring extreme bias. Finally, the quiescent, planar ITO configuration limits adhesion and mass transfer, allowing interfacial nuclei to detach and settle. This accounts for the significant reduction in dissolved (<0.2 µm) Cd observed in Sections 3.2 and 3.3 without formation of a thick electrode-bound film.

### 3.5. Practical implications and outlook

Applying a renewable PV bias enabled substantial cadmium removal from the dissolved fraction (62.3% over 7 days) under mild conditions with minimal chemical inputs, while producing recoverable Cd-bearing solids on both the cathode and in the catholyte. Two strategies emerge for practical optimization:

(1) Electrode design: transitioning from planar ITO to high-surface-area cathodes, such as porous carbons, foams, or textured oxides, could increase productivity and improve particle retention.

(2) Hydrodynamic optimization: flow-by or flow-through configurations can reduce boundary layer thickness and minimize detachment losses.

If metallic cadmium is the target product, stronger cathodic polarization, enhanced mass transfer, and controlled oxygen availability to limit local alkalization would favor plating over oxide or hydroxide formation. If oxide phases are acceptable, the current PV-assisted operation provides a viable route for cadmium capture and concentration suitable for downstream processing.

Recent studies have increasingly explored electrochemical and bioelectrochemical approaches for heavy-metal removal and recovery, emphasizing the importance of cathodic reaction pathways, competing side reactions, and mass-transfer limitations in determining product form (metallic deposition *vs.* precipitated solids). In particular, cadmium is challenging to recover as metallic Cd under low driving-force conditions, and many systems report precipitation-dominated immobilization unless sufficiently cathodic polarization is provided. Our results are consistent with this broader trend: PV assistance increased the effective driving force and promoted formation of recoverable Cd–O solids rather than continuous Cd plating, as supported by SEM/EDS/XRD and electrochemical evidence. Compared with conventional electrochemical treatment routes that often require higher applied voltages and/or harsh electrolyte conditions, the present PV-BES demonstrates a mild, renewable-biased operational window that favors solid-phase recovery under near-neutral bulk pH.^28,29^ These observations align with recent BES-focused reviews highlighting cathode material selection, biasing strategy, and hydrodynamics as primary levers for improving recovery performance and scale-up feasibility.^30^

Future studies could benchmark this system performance more rigorously by combining phase identification with current–voltage monitoring, performing full mass balances distinguishing electrode-bound, settled, and residual fractions, and reporting coulombic efficiency and energy intensity (kWh per kg Cd) under various operating modes. Even within the conservative, quiescent configuration tested here, these results demonstrate that solar-powered BES can integrate cadmium remediation with resource recovery, eliminating the need for harsh pH control or chemical dosing and providing a practical pathway toward sustainable metal management. A quantitative evaluation of energy efficiency and process economics was beyond the scope of this proof-of-concept study and will be addressed in future work as the system is scaled and optimized. In real wastewater matrices, competing ions, buffering capacity, and organic matter may influence cadmium speciation and precipitation behavior, and these matrix effects warrant further investigation under complex feed conditions.

## 4 Conclusion

This study demonstrates that a solar-powered bioelectrochemical system (BES) can remediate cadmium-bearing water under mild conditions while converting dissolved Cd^2+^ into recoverable solids. In seven-day batch experiments, the solar-assisted mode reduced the filtered-supernatant Cd^2+^ concentration from 1023.0 ± 11.1 mg L^−1^ to 385.99 ± 1.15 mg L^−1^ (62.3% removal), outperforming the self-biased MFC (53.8%) and the open-circuit control (30.4%). SEM and EDS analyses revealed sparse, island-like deposits on the ITO cathode containing Cd and substantial oxygen, while XRD of catholyte-collected particles confirmed the presence of crystalline Cd–O phases. These results indicate that cadmium immobilization was dominated by precipitation, followed by detachment and settling rather than continuous metallic plating. Mechanistically, the photovoltaic bias provides additional cathodic overpotential and localized interfacial alkalization, shifting conditions to favor Cd^0^ and Cd-oxy(hydroxide) formation over the dissolved Cd^2+^ fraction. Within the planar-ITO, quiescent configuration, this bias window enabled efficient removal without the need for harsh chemical inputs. Overall, solar-powered BES offers a renewable, low-chemical approach that integrates cadmium remediation with resource recovery. Future scale-up should focus on high-surface-area cathodes and flow-by or flow-through hydrodynamic designs to enhance productivity and retention of recovered Cd-bearing solids. By identifying a renewable-biased operational window that favors precipitation-dominated cadmium recovery under near-neutral bulk pH, this study provides mechanistic insight and practical guidance that extend beyond conventional electrochemical treatments requiring higher energy input or harsh pH control. Compared with previously reported Cd recovery systems, the PV-assisted BES achieved substantial dissolved-phase Cd removal (62.3% over 7 days) under mild conditions, highlighting its potential as a low-energy platform for sustainable metal remediation.

## Author contributions

Yeonu Im: investigation, formal analysis, data curation, writing – original draft, Minsoo Kim: writing – original draft, formal analysis, visualization, Yuri Kim: formal analysis, data curation, Soo Youn Lee: formal analysis, data curation, writing – review & editing, Jinhee Heo: formal analysis, data curation, writing – review & editing, Minkyoung Kim: formal analysis, writing – review & editing, Tae-Hoon Kim: formal analysis, writing – review & editing, Changman Kim: conceptualization, methodology, data curation, writing – original draft, writing – review & editing, supervision, project administration, funding acquisition.

## Conflicts of interest

The authors assert having no known competing financial interests or personal relationships likely to influence the work detailed in this paper.

## Supplementary Material

RA-016-D5RA07856C-s001

## Data Availability

The datasets produced and analyzed in this study can be obtained from the corresponding author upon reasonable request. Supplementary Information (SI) is available and includes raw and processed cadmium concentration data, XRD datasets, and SEM images of pristine and cadmium-deposited ITO electrodes. See DOI: https://doi.org/10.1039/d5ra07856c.
