# Correction: Solar-powered bioelectrochemical system for efficient cadmium remediation and recovery of reusable solids

**DOI:** 10.1039/d6ra90051h

**Published:** 2026-05-28

**Authors:** Yeonhu Im, Minsoo Kim, Yuri Kim, Soo Youn Lee, Minkyoung Kim, Tae-Hoon Kim, Jinhee Heo, Changman Kim

**Affiliations:** a Department of Biotechnology and Bioengineering, Chonnam National University Gwangju 61188 Republic of Korea cmkim@jnu.ac.kr +82 62 530 1949 +82-10-4566-2661; b School of Chemical Engineering, Pusan National University Busan 46241 Republic of Korea; c Gwangju Bioenergy R&D Center, Korea Institute of Energy Research (KIER) Gwangju 34129 Republic of Korea; d Department of Oceanography/KNU G-LAMP Project Group, Kyungpook National University Daegu 41566 Republic of Korea; e Department of Earth Systems and Environmental Sciences, Chonnam National University Gwangju 61188 Republic of Korea; f Advanced Characterization & Analysis Research Group, Korea Institute of Materials Science (KIMS) Changwon 51508 Republic of Korea; g Institute of Synthetic Biology for Carbon Neutralization, Chonnam National University Gwangju 61188 Republic of Korea

## Abstract

Correction for ‘Solar-powered bioelectrochemical system for efficient cadmium remediation and recovery of reusable solids’ by Yeonhu Im *et al.*, *RSC Adv.*, 2026, **16**, 2915–2922, https://doi.org/10.1039/D5RA07856C.

The authors regret that the funding information was incorrectly shown in the acknowledgements section of the original manuscript. The corrected funding acknowledgement is as shown below.

This work was financially supported by Chonnam National University (grant number: 2022-2547), the Research Development Project of the Gwangju Green Environment Center (No. 24-04-10-12-12), and the Basic Research Laboratory Program (BRL) by the National Research Foundation of Korea (NRF) grant funded by the Korea Government (MSIT) (RS-2025-02223550).

The Royal Society of Chemistry apologises for these errors and any consequent inconvenience to authors and readers.

