# Supplementary material for: Solar-powered bioelectrochemical system for efficient cadmium remediation and recovery of reusable solids
Source: RSC Adv. 2026 Jan 14;16(3):2915–22. doi: 10.1039/d5ra07856c (PMC12801247; doi:10.1039/d5ra07856c)
Supplement: RA-016-D5RA07856C-s001 [file RA-016-D5RA07856C-s001.pdf]

Supplementary Information (SI) for RSC Advances.
This journal is © The Royal Society of Chemistry 2026

Date: December 19, 2025

For submission to *RSC Advances*

Solar-powered bioelectrochemical system for efficient cadmium remediation and recovery of reusable solids

Yeonu Im[a,*], Minsoo Kim [b,*], Yuri Kim[a,c], Soo Youn Lee[c], Minkyoung Kim[d], Tae-Hoon Kim[e], Jinhee Heo[f] and Changman Kim[a,g]

[a] Department of Biotechnology and Bioengineering, Chonnam National University, Gwangju, 61188, Republic of Korea

[b] School of Chemical Engineering, Pusan National University, Busan, 46241, Republic of Korea

[c] Gwangju Bioenergy R&D Center, Korea Institute of Energy Research (KIER), Gwangju 34129, Republic of Korea

[d] Department of Oceanography /KNU G-LAMP Project Group, Kyungpook National University, Daegu 41566, Republic of Korea

[e] Department of Earth Systems and Environmental Sciences, Chonnam National University, Gwangju, 61188, Republic of Korea

[f] Advanced Characterization & Analysis Research Group, Korea Institute of Materials Science (KIMS), Changwon 51508, Republic of Korea

[e] Institute of Synthetic Biology for Carbon Neutralization, Chonnam National University, Gwangju, 61188, Republic of Korea

[*] The first two authors equally contributed to the manuscript.

Corresponding author

Prof. **Changman Kim**

Department of Biotechnology and Bioengineering,

Chonnam National University, Gwangju 61188, Republic of Korea

E-mail address: cmkim@jnu.ac.kr; Phone: +82-10-4566-2661, Fax: +82.62.530.1949

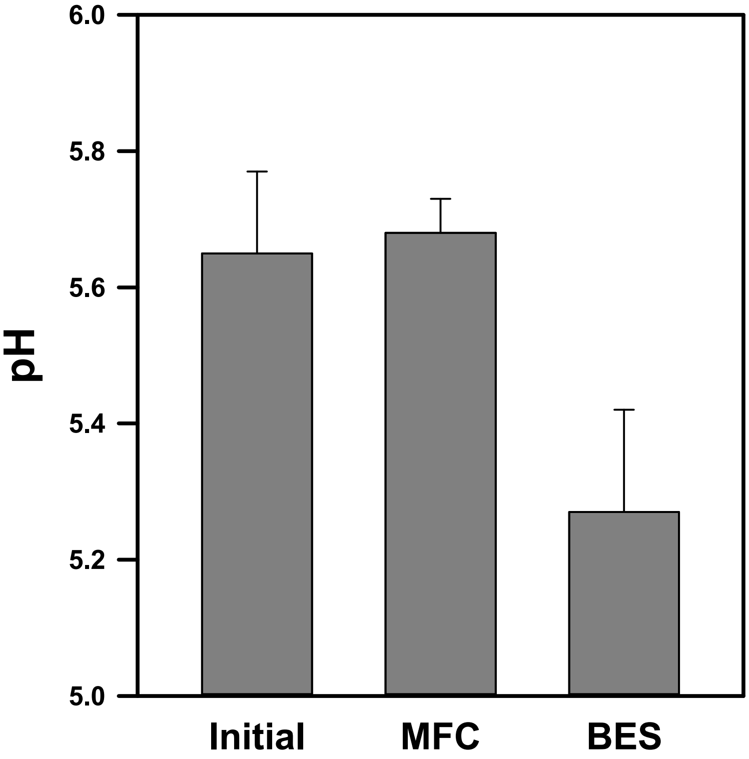

Fig. S1. Bulk pH of the catholyte before operation (Initial) and after 7 days under self-biased MFC and PV-assisted BES conditions (mean ± SD, n = 3).

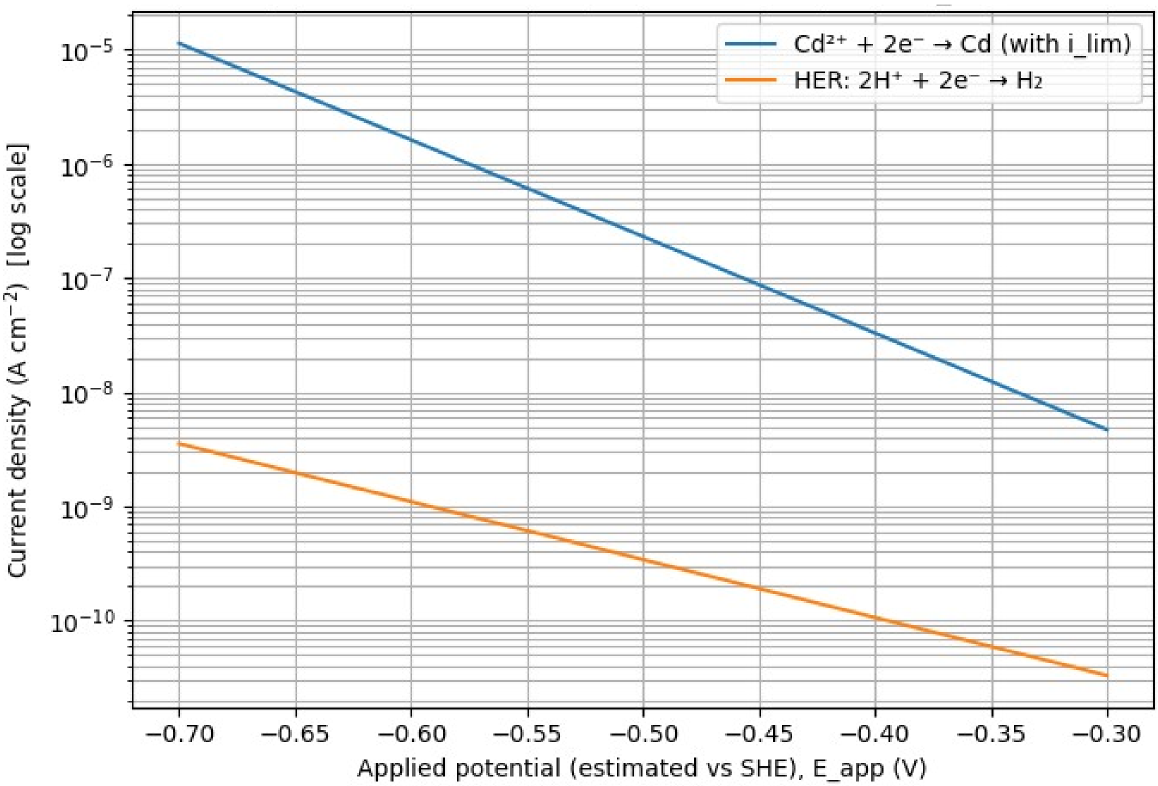

Fig. S2. Butler–Volmer estimates of cathodic competition between Cd^{2+} reduction ($Cd^{2+} + 2e^- \rightarrow Cd$, blue) and the hydrogen evolution reaction (HER, orange) at 25 °C. Calculations assume representative kinetic parameters for indium tin oxide (ITO) electrodes in mildly acidic conditions and include a diffusion-limited current density for Cd^{2+} reduction.
